# Sociocultural Influences on Smoking and Drinking

**Published:** 2000

**Authors:** Janet Kay Bobo, Corinne Husten

**Affiliations:** Janet Kay Bobo, Ph.D., is an epidemiologist in the Division of Cancer Prevention and Control and Corinne Husten, M.D., is a medical officer in the Office on Smoking and Health at the National Center for Chronic Disease Prevention and Health Promotion, Centers for Disease Control and Prevention, Atlanta, Georgia

**Keywords:** sociocultural AODC (causes of alcohol or other drug [AOD] use, abuse, and dependence), sociocultural aspects of AOD use, smoking, AOD use initiation, adolescent, family as an AODC, peer group, adult, AOD abstinence, public health

## Abstract

Numerous research studies have shown that sociocultural factors influence the initiation and continued use of alcohol and tobacco among adolescents and adults. Few studies have examined the effects of sociocultural factors on the tendency of smokers to drink and drinkers to smoke. However, the limited evidence available suggests that such factors exist and that the strength of the association between alcohol and tobacco use behaviors varies with the levels of alcohol use. Public health interventions focused on concurrent tobacco and alcohol use could yield further reductions in the morbidity and mortality associated with these substances.

Research indicates that sociocultural factors influence the initiation and continued use of alcohol and tobacco among adolescents and adults. Few studies have examined the effects of sociocultural factors on the tendency of smokers to drink and drinkers to smoke. However, the limited evidence available suggests that such factors exist and that the strength of the association between alcohol and tobacco use behaviors varies with the levels of alcohol use.

This article first reviews research on the sociocultural factors that influence whether adolescents begin smoking and/or drinking. The article then discusses similar mechanisms that may sustain alcohol and tobacco use among adults. Some sociocultural factors influence smoking and drinking across much of the adult population. Other factors that predict continued tobacco use among adults might differ for alcohol abstainers, moderate drinkers, heavy drinkers, and recovering alcoholics.

Although this article discusses adolescent and adult populations separately, a strong, positive relationship exists between alcohol and tobacco use in both age groups. Results from the 1997 National Household Survey on Drug Abuse (Substance Abuse and Mental Health Services Administration [SAMHSA] 1998) indicated that among the respondents ages 12 to 17 as well as ages 18 and older, current drinkers were much more likely to be current smokers compared with former drinkers or people who had never consumed alcohol (i.e., “never drinker”) (see table 1). The survey results also showed an especially strong relationship between binge drinking (defined as consuming five or more drinks per occasion) and current smoking among adolescents. Adolescents who reported engaging in binge drinking within the past 30 days were more than five times more likely to smoke than were adolescents who denied participating in binge drinking (76.8 versus 14.1 percent). Adults who reported episodes of binge drinking within the past 30 days were approximately twice as likely as were non-binge-drinking adults to be current smokers.

## Initiation and Continued Tobacco and Alcohol Use Among Adolescents

The strong association between binge drinking and smoking among adolescents may be attributable, in part, to the fact that both adolescent alcohol use and tobacco use share a number of sociocultural risk factors. Researchers have found that these factors—including family and peer influences, demographics, advertising, economics, and alcohol and tobacco availability—are associated with adolescents’ initial and continued tobacco and alcohol use.

### Family and Peer Influences

Adolescents are more likely to smoke cigarettes if their parents (Gritz et al. 1998; Flay et al. 1998), siblings (Mittelmark et al. 1987), or friends (Flay et al. 1998; Conrad et al. 1992) smoke. In addition, adolescents whose friends, siblings, or parents smoke are more likely to smoke at an earlier age than are other adolescents (Unger and Chen 1999). Adolescent alcohol use is also associated with drinking by peers (Botvin et al. 1998), parents (Brook et al. 1986), and siblings (Rittenhouse and Miller 1984). Together these findings demonstrate that family and peers exert similar influences on adolescent smoking and drinking.

Among teens, parent-child relationship factors—such as limited or poor-quality familial attachments; low levels of parental supervision and strictness; inadequate parental monitoring; and lack of parental affection, concern, and involvement—have also been related to smoking (Conrad et al. 1992; Biglan et al. 1995; Hundleby and Mercer 1987; Brook et al. 1983) and drinking (Arkin and Funkhouser 1990; Scaffa 1998). Data from the Adverse Childhood Experiences Study have further shown that any one of eight childhood stressors, including verbal, physical, or sexual abuse, significantly increases both a youth’s risk of smoking by age 14 and continued tobacco use as an adult (Anda et al. 1999).

### Demographic Factors

National student surveys show that white students, overall, are more likely than Hispanic and African-American students to report engaging in smoking (Kann et al. 1998; An et al. 1999) or drinking (SAMHSA 1998) within the previous 30 days. Hispanic students also are more likely to smoke (Kann et al. 1998) or drink (SAMHSA 1998) compared with African-American students. In addition, males generally are more likely than females to report current and frequent smoking (Kann et al. 1998) as well as current and heavy drinking (SAMHSA 1998).

In their review of multiple studies on teen smoking, Conrad and colleagues (1992) found that lower socioeconomic status consistently predicted smoking onset among teens. D’Onofrio (1997) found that alcohol use was also disproportionately concentrated among economically disadvantaged youth. Similar demographic factors have also been associated with adolescent smoking and drinking and may contribute to the relationship between smoking and drinking among adults.

### Advertising

In a study of adolescents who, when first interviewed, had never engaged in smoking, Pierce and colleagues (1998) found at the 3-year followup that having a favorite cigarette advertisement as well as possessing or being willing to use a tobacco promotional item at the initial interview predicted either future smoking or the increased likelihood of trying tobacco. Unger and Chen (1999) reported that the age of smoking initiation occurred earlier among adolescents who had a favorite tobacco advertisement, had received tobacco promotional items, or were willing to use tobacco promotional items. Research has also shown that awareness of beer commercials among fifth and sixth graders is significantly related to intentions to drink as adults (Grube and Wallack 1994), suggesting that alcohol advertising may influence adolescents to be more favorably disposed to drinking. Therefore, advertising has been found to be a potential risk factor for both smoking and drinking among adolescents.

### Economic and Availability Factors

Research suggests that adolescent smokers or drinkers are more likely than adults to reduce or quit smoking or drinking in response to increased cigarette or alcohol prices (see Grossman et al. 1993; Harris and Chan 1999). Harris and Chan (1999) found that 15- to 17-year-olds, compared with 18- to 20-year-olds, were more likely to respond to an increase in cigarette prices by quitting smoking rather than by reducing the number of cigarettes they smoked per day. Grossman and colleagues (1993) reported price elasticities of demand for cigarettes of −1.20 for 12- to 17-year-olds and −0.15 for people older than age 35, meaning that a 10-percent increase in the price of cigarettes would lower per capita consumption for these two populations by 12 and 1.5 percent, respectively. Other research suggests that a 10-percent increase in cigarette prices would reduce the number of teens who smoke by 7 percent (Grossman and Chaloupka 1997).

Alcohol use by young people is also more sensitive to price than drinking among adults. In one study, the price elasticity of demand for heavy drinking was −0.92 for people age 18 and older, compared with −2.24 for those between ages 18 and 21. The authors suggest that adults respond more to increases in the potential future costs or consequences of smoking and drinking, whereas young people are more sensitive to increases in price, because future costs are less important to them and young people have more stringent budget constraints than do adults (Grossman et al. 1993).

College students, however, may be less responsive to alcohol prices than are other groups of young people. Chaloupka and Wechsler (1996) examined drinking data from 17,000 college students in relation to beer prices and drunk driving laws in the locations of the participating colleges. The results suggested that college students were less responsive to alcohol prices than were other groups but that more severe drunk driving penalties tended to reduce both drinking and binge drinking. These effects were found among underage and older students, both male and female.

In another study that used data from multiple sources, including surveys of high school seniors conducted as part of the Monitoring the Future (MTF) project, researchers found a negative relationship between alcohol prices and both alcohol use and motor vehicle accident mortality and a positive relationship between college completion rates and alcohol prices. The researchers concluded that increases in Federal taxes on alcoholic beverages are effective in reducing youth alcohol consumption and alcohol-related injuries and deaths (Grossman et al. 1995). In fact, Chaloupka and colleagues (1993) reported that if the Federal excise tax on beer had been indexed to the rate of inflation since 1951, about 1,660 fewer 18- to 20-year-olds would have been killed in motor vehicle accidents in a typical year between 1982 and 1988. These studies show that adolescents are sensitive to increases in both tobacco and alcohol prices and that they are generally more sensitive to price than are adults.

The ease of obtaining cigarettes also influences smoking among adolescents. Both the general availability of cigarettes and offers of cigarettes from parents and siblings have been found to predict smoking onset among teens (Conrad et al. 1992). Vending machines increase the availability of tobacco products to youth and are used more often by children and adolescents than by the general public (U.S. Department of Health and Human Services [USDHHS] 1994).

## Tobacco and Alcohol Use Among Adults

Marketing practices related to the sale of alcohol and tobacco products, such as advertising, pricing, and availability, have been shown to influence the use of these substances among adults. Alcohol and tobacco advertisements depict drinking and smoking as fun activities that people do to relax and socialize. Advertising has been found to influence consumption among adults as well as youth. A meta-analysis of 48 studies that examined cigarette sales in relation to advertising and other factors, such as price, found that national spending on tobacco advertising was significantly related to tobacco sales (Andrews and Franke 1991). In another study that used data from a national survey of adults, researchers estimated that a 50-percent increase in the price of cigarettes could cause a 12.5-percent decrease in cigarette use (Farrelly and Bray 1998). The findings suggest that younger smokers would be more likely than older smokers to quit smoking in response to a price increase and that Hispanic smokers and African-American smokers would be more likely than white smokers to quit. In their review, Chaloupka and colleagues (1998) concluded that increased alcohol taxes and prices generally result in decreased alcohol consumption and related problems. In addition, they reported that research has shown a strong positive relationship between the increased availability of alcoholic beverages and the consequences of alcohol use and abuse.

Restrictions on the sale and use of alcohol have had complex and controversial effects on alcohol use. Although some groups continue to promote tighter restrictions on use, others argue that the Prohibition (1919–1933) amply demonstrated the ineffectiveness of legal bans on drinking (Morgan 1992).

Research suggests that workplace smoking bans and other restrictions on where smoking is allowed, however, may reduce smoking prevalence among people subjected to them. In a review of studies of workplace smoking restrictions, Chapman and colleagues (1999) reported that daily smoking rates declined in 18 of 19 studies and that smoking prevalence declined in 17 studies. Both workplace and household smoking restrictions have also been associated with higher rates of quit attempts, lower rates of relapse among smokers who attempted to quit, and higher rates of light smoking among current daily smokers (Farkas et al. 1999). Another study compared changes in smoking prevalence among smokers employed at smoke-free hospitals with changes in smoking among smokers in the community who were employed at non-smoke-free workplaces.^1^ Beginning with the smoking ban and continuing for 5 years after implementation, statistically significant differences in the postban quit ratio were observed between smokers employed at the smoke-free hospitals and their counterparts in the community. The overall difference in postban quit ratios remained significant even after adjustment for socioeconomic, demographic, and smoking-intensity variables (Longo et al. 1996).

The extent of tobacco and alcohol use may also be influenced by stress. One theory suggests that people are more likely to use drugs, especially multiple drugs, when unable to cope with stressful situations (Wills and Shiffman 1985). A 1995 review of the literature concluded that adults often use alcohol and tobacco for similar reasons, but the coping functions for alcohol use are more likely to involve distraction and forgetting, whereas the coping functions for smoking were related to increased attention and concentration (Wills and Cleary 1995).

A recent review of the “smoke to cope” literature portrays a more complex relationship between stress and smoking (Parrott 1999). Although most smokers report that cigarettes help them relax, several studies indicate that smokers are more anxious overall than are nonsmokers and that former smokers who maintain complete nicotine abstinence for at least 6 months report significant reductions in their stress levels. These findings suggest that nicotine-dependent smokers must smoke on a regular basis to cope with the withdrawal symptoms experienced when nicotine blood levels drop between cigarettes. Smoking is thus a major contributor to stress and a conditioned response to adverse moods.

Although much has been learned about sociocultural influences on adult tobacco use, the available literature does not satisfactorily explain the substantial variability in prevalence of smoking within the alcohol use levels displayed in table 1. Some insights on possible factors associated with this variability can be obtained by considering various levels of alcohol use sequentially.

## Tobacco Use Among Adult Lifetime Alcohol Abstainers

Adults who have rarely or never consumed 12 or more drinks per year have tobacco-use patterns that differ markedly from moderate drinkers, heavy drinkers, and recovering alcoholics or problem drinkers. As the data in table 1 indicate, smoking is relatively uncommon among nondrinkers. Factors that discourage tobacco use in this group have not been studied extensively to date, although the tendency for positive health behaviors to cluster in individuals is well established. In addition, several religious groups, including Mormons and Seventh Day Adventists, strongly oppose the use of both alcohol and tobacco (Jensen et al. 1996).

## Tobacco Use Among Adult Moderate Drinkers

The U.S. Government has defined moderate drinking as no more than one drink^2^ per day for women and no more than two drinks per day for men (USDHHS and U.S. Department of Agriculture [USDA] 1995). Similar to findings among adolescents, the sociocultural factors responsible for higher rates of tobacco use among adult moderate drinkers are poorly understood. Like adolescents, moderate drinkers may receive more frequent cues to smoke than do non-drinkers if many of their peers, family members, and social acquaintances also drink and smoke. These cues, along with the addictive nature of tobacco, may encourage continued smoking and compromise efforts to quit.

Research with smokers who drink suggests that drinking prompts smoking (Shiffman and Balabanis 1995). Participants in one study used small portable computers to record when they smoked and what else they were doing at the time (Shiffman et al. 1994). The computers also randomly “beeped” the participants to determine what they were doing during periods when they were not smoking. The participants were almost twice as likely to report recent drinking when they had been smoking**.** The results of this study are consistent with the theory that drinking influences smoking by releasing inhibitions that restrain smoking (Shiffman and Balabanis 1995).

## Tobacco Use Among Adult Heavy Drinkers

Smoking is especially prevalent among heavy drinkers, including people diagnosed with alcohol abuse or alcoholism.^3^ Table 2 summarizes the results of six studies published from 1967 to 1993 that reported the prevalence of tobacco use among alcoholics recently admitted for treatment. Until the early 1990s, about 90 percent of all such patients were regular smokers. More recent data suggest a marked decline in smoking prevalence in this population. Studies reported in 1996 (Hurt et al. 1996) and 1997 (Stuyt 1997) reported tobacco use rates among alcohol treatment patients of 75 and 71 percent, respectively.

Stress may influence levels of both smoking and drinking among heavy drinkers, as it does among moderate drinkers (Wills and Shiffman 1985). In addition, depression has been associated with smoking and severity of nicotine dependence (Ziedonis et al. 1998) and with alcohol dependence, although research has not determined whether alcohol dependence precipitates depression or results from it (see Schuckit 1996).

In a national survey of adults, smoking and drinking were both associated with self-reported negative moods (e.g., depression, loneliness, restlessness, boredom, and feeling upset) (Schoenborn and Horm 1993). Women who scored the highest on a scale of negative moods were almost three times more likely to smoke than were women who had a score of zero. Among men, the odds of smoking also increased with negative mood scores. Women with high negative mood scores were not significantly more likely to drink heavily (i.e., to consume an average of two or more drinks per day). However, among men, the odds of being a heavy drinker (i.e., of consuming an average of three or more drinks per day) more than tripled for those with the highest levels of negative moods compared with men with no negative moods. Negative moods also were associated with combined smoking and drinking. Men with the highest negative mood scores were four times more likely to combine smoking and heavy drinking than were men with no negative moods. The authors concluded that emotional health status and addictive behaviors were “sufficiently related to warrant increased public health initiatives that attempt to address both issues together rather than one at a time” (Schoenborn and Horm 1993, p. 8).

## Tobacco Use Among Recovering Alcoholics

Although several studies have reported encouraging data on the ability of recovering alcoholics to quit smoking (Sobell et al. 1995; Bobo et al. 1986), randomized clinical trial data suggest that most smokers who receive intensive treatment for a history of alcohol abuse or alcoholism continue to smoke long after learning to control their drinking. In addition, many continue to smoke heavily. In a longitudinal study of 575 adult smokers who completed intensive residential treatment for alcohol problems in the Midwest in 1995, 92 percent were still daily smokers 12 months after discharge from treatment (Bobo 1997). About one-half (49 percent) smoked an average of one or more packs of cigarettes per day.

Historically, three sociocultural mechanisms have influenced continued smoking among recovering alcoholics and problem drinkers. The first of these can be attributed to the widespread impact of Alcoholics Anonymous (AA) on recovering alcoholics in the United States. In addition to its well-known 12-step program, AA uses a number of pithy aphorisms to guide everyday behavior. One of the most important of these is “ First things first.” AA teaches its members that their primary responsibility is to become and remain sober. Often, members are advised to avoid tackling new challenges, like quitting smoking, until they are confident about their ability to remain sober even when under additional stress. A popular book published by AA uses a tobacco-related anecdote to illustrate this principle (AA 1976). The story reviews the case of an alcoholic whose wife “nagged” him to quit smoking after he had successfully stopped drinking. Unfortunately, the reader learns, “ her intolerance finally threw him into a fit of anger,” which resulted in his becoming drunk (AA 1976, p. 135). This vignette was used for many years to justify cigarette smoking during AA meetings. Although nonsmoking AA meetings are now available in most communities, they were rare until the mid-1980s.

Tobacco use policies at alcohol and other drug treatment centers constitute a second sociocultural factor that likely influences smoking among recovering alcoholics and problem drinkers. A 1982 survey of alcohol treatment inpatient facilities in Washington State found that more than one-half (53 percent) of the treatment staff believed a recovering alcoholic should not be encouraged to quit smoking until he or she had been sober for at least 1 year (Bobo and Gilchrist 1983). About one-fourth (23 percent) of the treatment staff indicated that they did not believe an alcoholic should *ever* be encouraged to quit smoking. Opinions were strongly associated with a personal history of smoking and alcoholism. Staff members who identified themselves as recovering alcoholics and current smokers were far less likely than staff members who self-identified themselves as nonsmokers with no histories of alcohol problems to report that they had ever personally encouraged an alcoholic to quit smoking.

Paralleling the profound shift in attitudes toward smoking that occurred across most U.S. communities in the 1980s, a marked change in acceptance of tobacco use was evident in alcohol and other drug treatment facilities by the early 1990s. A 1991 survey of 771 treatment personnel in Nebraska found that only 3 percent of the treatment personnel actively discouraged patients or clients who wanted to quit smoking (Bobo et al. 1995). However, only 35 percent of the staff members said that they thought recovering alcoholics who smoked should be encouraged to quit early in their sobriety. The most common reason given for a reluctance to encourage smoking cessation was the concern that the stress of trying to quit might adversely affect the patient’s ability to remain sober.

To address this concern, during 1995 researchers conducted a randomized community intervention trial in 12 residential treatment facilities in Iowa, Kansas, and Nebraska (Bobo et al. 1998). Patients in one-half of the treatment centers received a four-part intervention to encourage smoking cessation. Patients in the remaining six centers (i.e., the control group) received the usual care provided by those facilities. The results of the study clearly showed that alcoholic patients who smoked could benefit from smoking cessation counseling. After 1 year of followup, 43 percent of the patients who were encouraged to quit smoking were still abstaining from alcohol, compared with 29 percent of the patients in the control centers. These findings do not suggest that smoking cessation efforts among recovering alcoholics will be free of stress, but they do indicate that alcohol treatment facilities can safely provide a socio-cultural climate that explicitly encourages smoking cessation. Findings from other studies have shown that instituting smoking cessation programs in alcohol treatment centers is acceptable to treatment staff and does not adversely affect patient enrollment (Hurt et al. 1994; Joseph et al. 1993; Patten et al. 1999).

Evidence of a third sociocultural influence on tobacco use among recovering alcoholics and problem drinkers was suggested by an unanticipated finding in the 12-site randomized community intervention trial previously described. Two of the twelve sites (one tobacco intervention site and one control group site) restricted admission to their programs to alcoholics who were either Native American or Alaska Native. The smoking cessation rates among patients in these programs were nearly twice as high as those observed among all other racial and ethnic groups in the study (Bobo 1997). Treatment staff in the Native American programs attributed the difference in quit rates to frequent discussions with their patients about the sacred role of tobacco in many Native American communities and its traditional use in various religious ceremonies.

## Public Health Implications

The information reviewed in this article on the sociocultural influences on smoking and drinking suggests several strategies that health care providers and public health practitioners could use to discourage alcohol and tobacco use and alcohol abuse. In addition to ongoing efforts to restrict adolescent exposure to tobacco and alcohol advertising and discourage sales of these products to minors, intervention efforts targeted toward the well-established association between smoking and drinking could prove beneficial. People with histories of problem drinking should be screened for tobacco use, informed of the added health risks of tobacco use for heavy drinkers, and encouraged to quit smoking. New media campaigns to educate the general public on the additional dangers of smoking for moderate and heavy drinkers would also be appropriate. Dedicating some public health research dollars to the development of smoking cessation programs tailored to the needs and interests of recovering alcoholics would also be useful. The National Institute on Alcohol Abuse and Alcoholism has supported some work in this area, but many important clinical questions remain unanswered.

Other innovative strategies could be considered as well. State legislatures could be urged to pass laws prohibiting smoking and tobacco purchases in bars and taverns. The entertainment and advertising industries could be encouraged to avoid images of people smoking cigarettes in scenes portraying alcohol use and vice versa. And finally, alcohol treatment facilities could be instructed to completely ban all tobacco use on their premises and require all their employees to be nonsmokers.

## Conclusion

This article has reviewed key findings from the large body of literature on sociocultural mechanisms that encourage tobacco and alcohol use among adolescents and adults. Many of these mechanisms exert similar effects on both alcohol and tobacco use behaviors. Socio-cultural factors that encourage smokers to drink and drinkers to smoke have not received extensive study, but they may account for some of the substantial variation in adult tobacco use rates seen among different levels of alcohol consumption.

Although studies based on samples of problem drinkers and recovering alcoholics suggest a recent weakening of the association between drinking and smoking that is consistent with changes in societal attitudes toward tobacco and standards of care in alcoholism treatment facilities, the connection may continue to be quite strong among some populations (e.g., polydrug users and depressed adults). “ Hard-core” smokers and drinkers may particularly benefit from additional research on sociocultural mechanisms that strengthen the association between these behaviors. Such research may identify new opportunities for treatment and prevention and promote changes in public health policy that would further discourage concurrent use of tobacco and alcohol.

## Figures and Tables

**Table 1:** Tobacco Use Among Four Categories of Adolescent and Adult Alcohol Users in the General Population

Alcohol Use History	Current Smoker (%)	Former Smoker (%)	Never Smoker (%)
**Ages 12 to 17**			
Current drinker	58.1	23.4	18.5
Former drinker	23.8	37.9	38.3
Never drinker	05.6	11.2	83.2
Binge drinking* in past 30 days			
Yes	76.8	17.9	05.3
No	14.1	18.8	67.2**
**Age 18 and older**			
Current drinker	36.9	45.6	17.5
Former drinker	27.1	50.8	22.1
Never drinker	13.4	18.8	67.9**
Binge drinking in past 30 days			
Yes	54.5	35.7	09.9**
No	26.1	45.1	28.7**

* Binge drinking was defined as five or more drinks on one occasion.

** Values shown are from weighted analyses. Due to rounding, some row totals will not sum to exactly 100.

SOURCE: Substance Abuse and Mental Health Services Administration 1997.

**Table 2:** Prevalence of Smoking in Various Samples of Adults With Histories of Heavy Drinking

Year	Authors	Sample (*n*)	Smokers (%)	
1967	Dreher and Fraser	103 alcoholics in outpatient treatment	97 (males)	91 (females)
1972	Walton	130 male alcoholics in treatment	97 (males)	
1986	Kozlowski et al.	1,142 alcoholics in treatment in Canada	86 (males)	82 (females)
1989	DeSoto et al.	312 alcoholics recruited from Alcoholics Anonymous	98 (males)	92 (females)
1990	DiFranza and Guerrera	77 alcoholics in treatment	83 (combined)*	
1993	Joseph et al.	176 male alcoholics admitted before implementation of tobacco control policy in the facility	82 (males)	
1995	Batel et al.	325 male and female outpatients in an alcohol treatment program	88 (combined)*	
1996	Hurt et al.	845 alcohol treatment patients	75 (combined)*	
1997	Stuyt	174 alcohol treatment patients	71 (combined)*	

* Data were not reported separately by gender.

## Footnotes

1 The authors contend that hospital employees comprise an appropriate study population and cite evidence that hospital employees smoke at rates equivalent to people in the general population. The study excluded physicians and included managerial, clerical, blue-collar, skilled, and unskilled workers.

2 A drink is considered to be 12 ounces of regular beer, 5 ounces of wine, or 1.5 ounces of 80 proof distilled spirits (USDHHS and USDA 1995).

3 Alcohol abuse and alcoholism are conditions defined according to specific criteria published by the American Psychiatric Association (APA) in its *Diagnostic and Statistical Manual of Mental Disorders* (DSM). The most recent edition (DSM–IV) defines alcohol dependence as a cluster of symptoms that includes continued drinking despite significant alcohol-related problems. Alcohol abuse is defined as repeated drinking in harmful situations with negative consequences (APA 1994).
